# *In silico* annotation of a hypothetical protein from *Listeria monocytogenes* EGD-e unfolds a toxin protein of the type II secretion system

**DOI:** 10.5808/gi.22071

**Published:** 2023-03-31

**Authors:** Maisha Tasneem, Shipan Das Gupta, Monira Binte Momin, Kazi Modasser Hossain, Tasnim Binta Osman, Md. Fazley Rabbi

**Affiliations:** Department of Biotechnology and Genetic Engineering, Noakhali Science and Technology University, Noakhali 3814, Bangladesh

**Keywords:** hypothetical protein, *in silico* characterization, *Listeria monocytogenes*, PemK/MazF-like toxin, three-dimensional structure, type II toxin-antitoxin system

## Abstract

The gram-positive bacterium *Listeria monocytogenes* is an important foodborne intracellular pathogen that is widespread in the environment. The functions of hypothetical proteins (HP) from various pathogenic bacteria have been successfully annotated using a variety of bioinformatics strategies. In this study, a HP Imo0888 (NP_464414.1) from the *Listeria monocytogenes EGD-e* strain was annotated using several bioinformatics tools. Various techniques, including CELLO, PSORTb, and SOSUIGramN, identified the candidate protein as cytoplasmic. Domain and motif analysis revealed that the target protein is a PemK/MazF-like toxin protein of the type II toxin-antitoxin system (TAS) which was consistent with BLASTp analysis. Through secondary structure analysis, we found the random coil to be the most frequent. The Alpha Fold 2 Protein Structure Prediction Database was used to determine the three-dimensional (3D) structure of the HP using the template structure of a type II TAS PemK/MazF family toxin protein (DB ID_AFDB: A0A4B9HQB9) with 99.1% sequence identity. Various quality evaluation tools, such as PROCHECK, ERRAT, Verify 3D, and QMEAN were used to validate the 3D structure. Following the YASARA energy minimization method, the target protein's 3D structure became more stable. The active site of the developed 3D structure was determined by the CASTp server. Most pathogens that harbor TAS create a crucial risk to human health. Our aim to annotate the HP Imo088 found in *Listeria* could offer a chance to understand bacterial pathogenicity and identify a number of potential targets for drug development.

## Introduction

*Listeria monocytogenes* is a pathogenic facultative, intracytosolic, gram-positive bacterium for humans and several animal species which is responsible for the infection-listeriosis [1]. Listeric infections are often associated with gastrointestinal (GI) listeriosis (non-invasive) in immunocompetent persons or listeriosis (invasive) in immune-compromised individuals [2,3]. Invasive *listeriosis* is responsible for meningitis in immunocompromised people and miscarriage in pregnant women [4]. Patients with chronic renal failure and cirrhosis, as well as those on drugs to reduce gastric acidity, are at greater risk of listeriosis [5]. Moreover, urinary tract infections by *L. monocytogenes* were also recorded, in an instance after detecting this bacterium in urine samples [6]. One of the prime reasons for listeriosis outbreaks is inadequate hygiene standards and insufficient implementation of standard sanitation operating procedures in the food manufacturing industry [7]. Multidrug-resistant *L. monocytogenes* has been found in irrigation waters and agricultural soils, and can spread to agronomical fresh product risking food safety [8]. There is compelling evidence that contaminated food, mostly ready-to-eat meals, is the main route of transmission of this bacterium into humans. The ability of *L. monocytogenes* to traverse host barriers (such as the intestinal barrier, and the materno-fetal barrier), is responsible for causing listeriosis. *L. monotocytogenes* can also be detectable in the bloodstream during an infection [9]. After successfully evading the GI tract environment, *L. monocytogenes* can invade and persist in mammalian host cells due to presence of multiple virulence factors [10]. Because of the clinical significance of listeriosis, several genomes of *L. monocytogenes* strains have already been sequenced to have a deeper understanding of the species' lifestyle and pathogenicity, as well as the possible impact of strain variability on virulence. *L. monocytogenes* could be divided into four evolutionary lineages and four serogroups based on polymerase chain reaction testing in the two genes of hlyA and iap [11,12]. The major portion of *L. monocytogenes* isolates are from lineage I, including serotypes 1/2b, 3b, and 4b. On the other hand, lineage II comprises serotypes 1/2a, 1/2c, 3a, and 3c. The bacteria *L. monocytogenes* EGD-e (serovar 1/2a) is responsible for various listeriosis outbreaks. The EGD-e strain has a circular chromosome consisting of 2,944,528 bp with an estimated 39% of G + C content [13]. The EGD-e strain of *L. monocytogenes* genome has been predicted to contain a sum of 2,853 genes that code for proteins. A large number of genes that regulate the pathogenicity, development, and survival of EGD-e strain have already been characterized by researchers and annotated in the published genome sequence [14-17]. Nonetheless, numerous loci with putative genes that codes for protein are designated as "Hypothetical proteins (HP)" because the relationship between those proteins and listeria life cycle are poorly understood. An HP is predicted to be encoded by a recognized open reading frame but has no experimental evidence to support its putative function [18]. In most genomes, around 50% of the protein-coding genes are designated as HPs. Thereby, HPs are likely to have their own significance in an organism's overall proteomic platform. An appropriate annotation of the HPs found in a pathogen helps researcher not only to gain an improved knowledge of the pathogen's virulent actions but also to discover new structures of proteins, metabolic pathways, and functions [19]. HPs may likely to act an important role in organisms’ growth, survival, and disease progression. They can also serve as potential pharmacological targets and genetic markers for the development of novel antimicrobial medicines and medications [20]. In recent times, bioinformatics has improved our knowledge of protein function-structure interactions. Bioinformatics analysis has the advantage of being less expensive and time-saving than conventional *in vitro* procedures. Functional annotation of HPs utilizing different structural and sequence-specific bioinformatics softwares can aid in the classification of these proteins into which several functional groups, providing more information about their structures, activities, and contribution in metabolism [21]. Because *L. monocytogenes* is prevalent in surroundings, constant focus by risk managers is much needed to control *Listeria* in food production facilities. Therefore, to curtail the prevalence and to develop effective control measures against listeriosis, a better understanding of the microorganism's characteristics, environmental influence, and host-virulent factors interactions are required [22]. Thus, the main objective of this study is to ascribe a potential biological function and predictive structure to the HP Imo088 (accession No. NP_464414.1) of *L. monocytogenes EGD-e*. The protein sequences were analyzed utilizing latest bioinformatics software and tools for homology search against functionally characterized proteins, determination of domain and physicochemical properties, prediction of subcellular localization, and determination of active site. We believe that this interpretation will strengthen our knowledge about the functional activities of the HP Imo088 found in listeria and provide a platform to discover potential pharmacological targets.

## Methods

### Sequence retrieval with FASTA format

The FASTA sequence of the HP (NP_464414.1) were obtained from the NCBI (National Center for Biotechnology Information; https://www.ncbi.nlm.nih.gov/) [23] database. The protein sequence was then submitted on to numerous prediction servers for the in-silico annotation [24].

### Physicochemical properties analysis

The ExPASy ProtParam (https://web.expasy.org/protparam/) tool was used to characterize HPs in terms of their physicochemical features which includes molecular weight, aliphatic index, extinction coefficients, amino acid composition, grand average of hydropathy (GRAVY), isoelectric point (pI), and estimated half-life [25].

### Prediction of protein subcellular localization

The putative subcellular localization of the HP was determined by CELLO v.2.5 (http://cello.life.nctu.edu.tw/) [26], an analysis based on a two-level support vector prediction system. Subcellular localization predicted by CELLO was further correlated with the result of PSORTb (https://www.psort.org/psortb/) [27], SOSUI (https://harrier.nagahama-i-bio.ac.jp/sosui/mobile/) [28], and PSLpred (https://webs.iiitd.edu.in/raghava/pslpred/submit.html) [29]. SOSUI discriminates between soluble and transmembrane proteins by calculating the average hydrophobicity of protein. In contrast, PSORTb and PSLpred predict subcellular localization of prokaryotic proteins on the basis of various features e.g., amino acid and dipeptide composition, composition of 33 physicochemical properties, and evolutionary information of PSI-BLAST.

### Identification of protein domain and motif

For protein domain analysis, NCBI CD-Search (https://structure.ncbi.nlm.nih.gov/Structure/cdd/wrpsb.cgi) [30], Pfam 34.0 (http://pfam.xfam.org/) [31], InterProScan5 (http://www.ebi.ac.uk/Tools/services/web/toolform.ebi?tool=iprscan5&sequence=uniprot:KPYM_HUMAN). To determine the protein sequence motif, MOTIF Search (https://www.genome.jp/tools/motif/) tool was used [32]. Conserved domain (CD) search compares a query sequence with the CD alignments which was found in the Conserved Domain Database (CDD). The functional analysis of the protein was carried out by using the InterProscan tool. Pfam is a protein family database that uses hidden Markov models (HMMs) in order to generate annotations and multiple sequence alignments.

### Protein family and phylogenetic tree analysis

The homologs of the HP (NP_464414.1), a protein-BLAST (BLASTp) (https://blast.ncbi.nlm.nih.gov/Blast.cgi?PAGE=Proteins) [33] from NCBI (National Center for Biotechnology Information) against the non-redundant database with default parameters was performed. This approach is based on the local alignment of protein sequence to find similar proteins. CLC Sequence Viewer version 8.0 was used for multiple sequence alignment and to create phylogenetic tree for few selected sequences.

### Secondary structure prediction

Two-dimensional structure of the NP_464414.1 protein was determined using SOPMA (Self-optimized prediction method with alignment) (https://npsa-prabi.ibcp.fr/cgi-bin/npsa_automat.pl?page=/NPSA/npsa_sopma.html) [34] and PSIPRED (Position Specific Iterated – BLAST) (http://bioinf.cs.ucl.ac.uk/psipred/) [35]. Result from SOPMA analysis was correlated with the result of PSIPRED.

### Homology modeling

The Alpha Fold 2 Protein Structure Prediction Database (https://alphafold.ebi.ac.uk/) [36] was used to determine the 3D structure of our putative HP and the performance of this determination was based on the pairwise comparison profile of HMMs. The template protein of the type II toxin-antitoxin system PemK/MazF family toxin protein (DB ID_AFDB: A0A4B9HQB9) was retrieved from the query result for homology-based modeling. UCSF Chimera 1.16 was employed to visualize the 3D model structure.

### Quality assessment

To assess the quality of the predicted 3D structure, various evaluation tools were used. These include PROCHECK (https://www.ebi.ac.uk/thornton-srv/software/PROCHECK/), Verify3D (https://servicesn.mbi.ucla.edu/Verify3D/) [37], and QMEAN (https://swissmodel.expasy.org/qmean/) [38] programs of ExPASy server of SWISS-MODEL Workspace.

### Active site analysis

CASTp Computed Atlas of Surface Topography of proteins; http://sts.bioe.uic.edu/castp/) server was applied to predict and locate the protein’s active site. CASTp not only predict the active pockets residing in protein surfaces, but also the key residues and the regions of protein that interact with ligands in the inner region of the three-dimensional structure.

### Energy minimization of the model structure

The energy of the 3D model structure was minimized using YASARA (http://www.yasara.org/minimizationserver.htm) [39] force field minimizer. After refining through YASARA, a more stable and reliable 3D structure of the target protein was gained.

## Results

### Physicochemical properties

ProtParam tool was used to determine some crucial physiochemical features. The protein was predicted to contain 115 amino acids, an isoelectric point (PI) of 6.73, and a molecular weight of 12,759.85 Da. The calculated value of GRAVY of the protein was −0.057. The protein was classified as stable, because the instability index of the desired protein was computed to be 36.52. The most abundant amino acids were valine (11.3%), isoleucine (9.6%), leucine (8.7%), lysine (7.8%), asparagine (7.8%), alanine (7%), glutamate (6.1%), glycine (6.1%), threonine (6.1%), arginine (5.2%), glutamine (4.3%), tyrosine (1.7%), and histidine (1.7%). Surprisingly, tryptophan and cysteine were found to be completely absent in the protein sequence. The protein contains a total of 16 negatively charged (Asp + Glu) and 16 positively charged (Arg + Lys) amino acids (Table 1). The report on atomic composition showed that the protein comprises of 1839 atoms having molecular formula of protein C_568_H_943_ N_155_O_170_S_3_.

### Subcellular localization

A protein's function is significantly influenced by where it is located within the cell. For this reason, understanding a protein's location in the cellular setting is beneficial to discover proteins with undetermined function. Determination of subcellular localization of the target protein was done by CELLO and further confirmed by PSORTb, SOSUIGramN, and PSLpred server. It was found by all of these methods that the protein would be cytoplasmic (Table 2). This knowledge might be helpful for interpreting the functional role as well as for designing a drug against the target protein.

### Domain and motif identification

The specific hit explored by CD search tool predicted that the query protein belongs to PemK toxin superfamily with an E-value of 2.87e−43. This domain covers 5 to 110 amino acid residues of our protein sequence (Table 3). The result of the CD search analysis was found to be comparable with the outcome of InterProScan and Pfam. The Pfam tool predicted the PemK toxin superfamily domain at 4 to109 amino acid residues. The InterProScan server predicted PemK-like domain in the range of 1 to 112 amino acid residues of the HP. Similarly, the MOTIF server predicted the PemK domain at the position of 4 to 109 amino acid residues having an E-value of 2.6e-33. PemK is a growth inhibitor that is found in *Escherichia coli* and it auto regulating synthesis by binding to the promoter region of Pem operon. A typical bacterial toxin-antitoxin system contains the toxin molecule of this family. A number of different toxins, such as MazF, Kid, PemK, ChpA, ChpB, and ChpAK are also members of this family [40].

### Protein family and phylogeny analysis

**Fig. 1.**
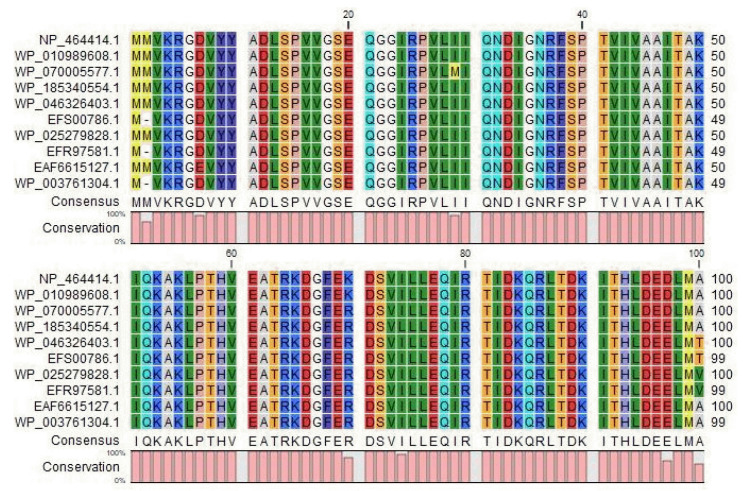
Multiple Sequence Alignment among different type II toxin-antitoxin system proteins with the target protein at the top row (sources for the sequences: Row 2, 3 and 9, *Listeria monocytogenes*; Row 4 to 6, *Listeria seeligeri*; Row 7 to 8, *Listeria ivanovii*, Row 10; *Listeria immobilis*). The figure was generated by CLC Sequence Viewer version 8.

**Fig. 2.**
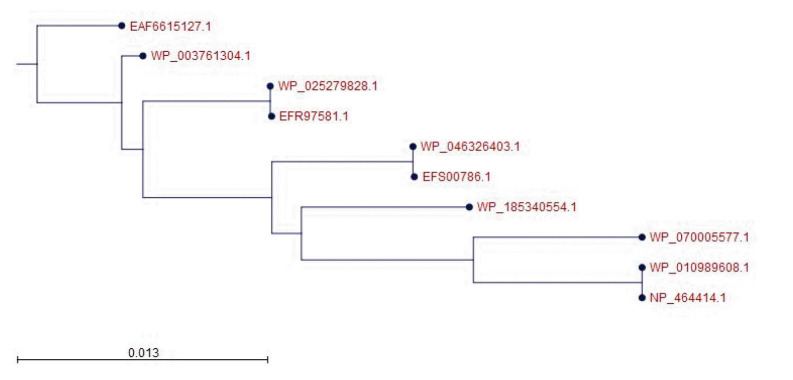
Phylogenetic tree with true distance from target protein (NP_464414.1). The tree was generated using CLC Sequence Viewer version 8. Here, the scale bar estimates sequence divergence, and amount of genetic change is represented by the line segment with the number (0.013).

The BLASTp search was carried out against the non-redundant database which showed sequence similarities (up to 96 %) with other known PemK/MazF family toxin proteins of type II toxin-antitoxin system from different *Listeriaceae* (Table 4). Multiple sequence alignments of few selected proteins retrieved from BLASTp results were done to observe the conserved and dissimilar residues among the homologs (Fig. 1). A phylogenic tree was built using the same information (Fig. 2). The target protein, as well as two other *Listeria monocytogenes* proteins, appear to have a common ancestor with the WP_185340554.1 protein of *Listeria seerigeli*. The scale bar estimates sequence divergence, and amount of genetic change is represented by the line segment with the number (0.013).

### Secondary structure prediction

**Fig. 3.**
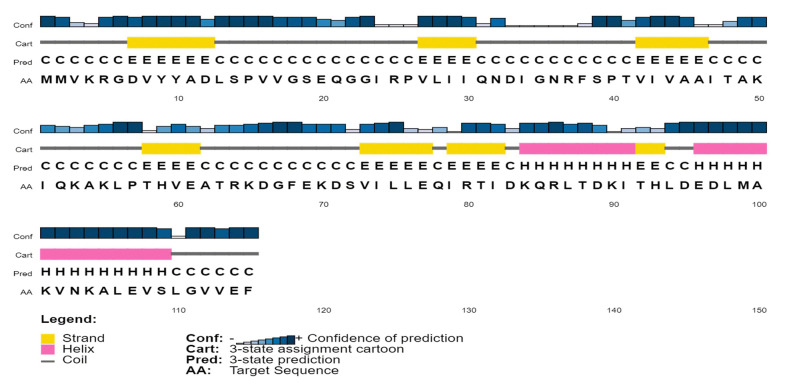
Predicted secondary structure of the target protein using PSI-PRED server. This graphical representation has four different sections. First section consists of bars with different heights. The length of the bar height is proportional to confidence score. In the second section, the pink color denotes the alpha helix, the yellow color denotes beta sheets or strands, and the gray color depicts coils; the coil connects a particular alpha helix with the particular beta sheets. The third section contains an alphabetic representation, which denotes the secondary structure of a protein; Here E, H, C are used for beta sheets, alpha helixes and coils, respectively. In the last section, the arrangement of amino acids is presented in alphabetic form.

To analyze the protein’s secondary structure, a server named SOPMA was used to estimate the proportions of extended strand (21.74%), alpha helix (33.91%), beta turn (3.48%), and random coil (40.87%). Similar outcomes were also found during PSIPRED analysis (Fig. 3).

### Three-dimensional structure determination and model quality assessment

**Fig. 4.**
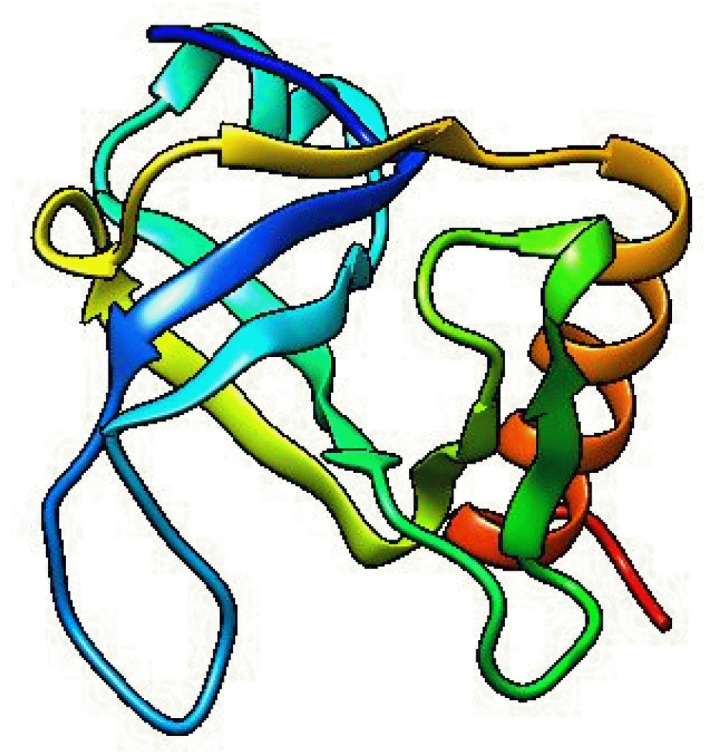
Predicted three-dimensional structure of the target protein (visualized by UCSF Chimera 1.16).

**Fig. 5.**
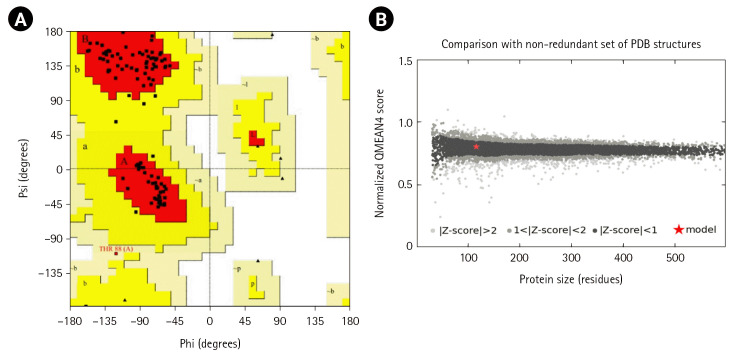
Model Quality Assessment. (A) Ramachandran plot of the model structure validated by PROCHECK server. Here, 92.2% amino acid residues covered the most favored regions [A, B, L]. (B) Graphical representation of QMEAN result of the model structure. Here, Z score of the anticipated model was 0.40 (indicates good agreement between the model structure and experimental structure of similar size).

The template structure of the type II toxin-antitoxin system PemK/MazF family toxin protein (DB ID_AFDB: A0A4B9HQB9) was used to determine the 3D structure of our target protein, which showed 99.1% identities with our desired protein in the Alpha Fold 2 Protein Structure Prediction Database. The template protein is a toxin MazF protein from *Listeria monocytogenes*. The structure was visualized by UCSF Chimera 1.16 (Fig. 4). PROCHECK was used to evaluate the projected 3D structure of target protein through Ramachandran plot analysis. As reported by PROCHECK, 92.2% amino acid residues covered the most favored regions in “Ramachandran plot” that is regarded as a valid model quality (Table 5, Fig. 5A). By Verify 3D plot, we concluded that 93.04% of the residues had an averaged 3D-1D score ≥ 0.2. The overall quality factor of the predicted protein came out to be 95.1923 through ERRAT program. The predicted model reliability is reflected through the QMEAN4 score which compares the model structure with already determined experimental structure of similar size. The QMEAN4 global score of our target protein is 0.40 which indicates as good (Fig. 5B).

### Active site analysis

**Fig. 6.**
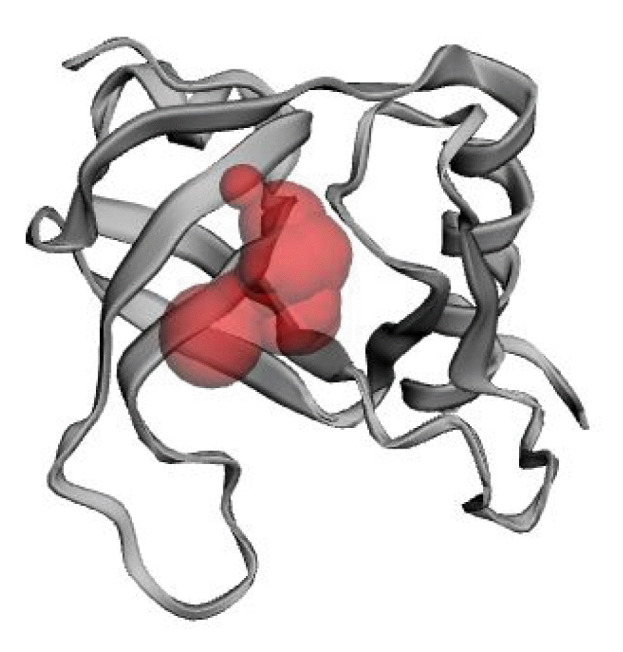
Determination of active site using CASTp server. The largest active site was found in the areas with 74.975 and volume of 24.651 amino acids.

By using the CASTp server, the active site of the developed 3D structure was assessed (Fig. 6). The most active site was discovered in one of the largest pockets with 74.975 solvent-accessible (SA) surface area and a total volume of 24.651 amino acids, respectively. Key active residues predicted from pocket are TYR^10^, ILE^24^, ILE^47^, THR^48^, ALA^49^, PHE^68^, ARG^70^, SER^72^, and ILE^91^. The main step when designing a medication or inhibitor is the identification of active site of amino acids.

### Energy minimization result

The energy of the protein’s three-dimensional structure was minimized by YASARA force field minimizer. The energy was reduced to –51,862.6 kJ/mol from –65,533.5 kJ/mol after energy minimization. The initial value was –0.04 kJ/mol; however, after the minimization process, the end value was 0.57 which indicates the structure as stable one.

## Discussion

*Listeria* is an intrinsic pathogen that is gram-positive, rod-shaped, non-spore producing and catalase positive. The *Listeria* genus contains 17 species, six of which *L. monocytogenes*, *L. ivanovii*, *L. seeligeri*, *L innocua*, *L. welshimeri*, and *L. grayi* are most frequent [41]. Among these species, only *L. monocytogenes* is responsible for serious complications in both human and animals. *L. monocytogenes* is a prominent cause of foodborne disease worldwide, with a high hospitalization and fatality rate. Characterization of HPs NP_464414.1 of *L. monocytogenes* EGD-e can aid in understanding bacterial metabolic regulations, formulating disease control strategies, and developing effective therapeutics. Various computational resources were employed in this study to characterize the HP NP_464414.1 of *L. monocytogenes* EGD-e from structural and functional aspects. The physiochemical properties’ analysis revealed that the protein consists of 115 amino acid sequence, have a molecular weight of 12,759.85, the GRAVY score of –0.057, and a theoretical PI of 6.37 (Table 1). In our investigation, we used CELLO for the prediction of subcellular location which revealed the query protein to be a cytoplasmic one. The analysis of the protein’s secondary structure reveals the prevalence of extended strand, beta turn, alpha helix, and random coil. Domain and motif study indicates that our target HP belongs to PemK toxin superfamily. A typical bacterial toxin-antitoxin system contains the toxin molecule of this family (Table 3). We used other bioinformatics resources to confirm that the prediction was highly accurate. BLASTp against the non-redundant database revealed up to 96 % sequence similarity with other type II toxin-antitoxin system PemK/MazF family toxin (*L. monocytogenes*) (Table 4). TASs are small genetic components composed of toxic protein and its antitoxin protein, with the latter counteracting the former's toxicity. Through *in-silico* analysis, two toxin-antitoxin systems (TASs) (lmo0113-0114 and lmo0887-0888) are found in *L. monocytogenes* EGD-e using TADB2. Only a few studies on TASs of *L. monocytogenes* have been conducted so far and those were also limited to few strains and few TAS pairs [42,43]. The strain ATCC19117 was studied using *in silico* approach where few TASs pair were found (lmo0887-0888, lmo0113-0114, and Imo1301-1302) with subsequent 3D structure and possible inhibitory peptide analysis [44]. As an endoribonuclease, the toxin PemK selectively identifies and cut the tetrad sequence UAUU in a target mRNA without the need for ribosomes. It is suggested that the antitoxin Pemk acts as both a transcription factor and a toxin activity neutralizer, enabling bacterial survival [45]. It's interesting to note that, unlike the cell translation machinery, the target sequence (UAUU) of PemK is present in a sizable percentage of mRNA transcripts that encode virulence related protein [46,47]. The regulated proteins were implicated in a variety of processes, including, cytoskeleton function, protein and lipid synthesis, heat shock and stress response, ATP synthesis, innate immunological defense, muscle construction, and others. The overexpression of the pemK gene severely inhibited bacterial growth in the case of other dangerous bacteria, such as *Mycobacterium tuberculosis*, *Klebsiella pneumonia*, and *Bacillus anthracis* [48]. PemK toxins coordinate the modulation of particular gene pools in the bacterial transcriptome, but their experimental characterization is challenging. The tertiary structure of the protein was developed from Alpha Fold 2 Protein Structure server and the quality of the model was assessed by evaluation software like Verify 3D, PROCHECK, ERRAT, and QMEAN. 92.2 % amino acid residues covered the most favored region in Ramachandran plot, which depicts the model quality as valid (Fig. 5A). The result of QMEAN4 server (Fig. 5B) revealed that the Z score of the anticipated model was 0.40, which also denotes a good quality model. After YASARA energy minimization process, the 3D structure of target protein became more stable which turned to be 0.57. In CASTp analysis, one largest pocket was found as active sites with SA surface area of 74.975 and volume of 24.651 amino acids. The majority of viruses with toxin-antitoxin in their system pose a significant threat to individual’s health [44]. Although tremendous progress has been made in investigating the roles of toxin-antitoxins in recent years, many functional and structural aspects of toxin-antitoxins and their effectors remain elusive. Our aim of the study was to identify the structural and biological function of the HP NP_464416.1 of *L. monocytogenes* through an *in silico* approach. This annotation of the HP is fundamental to strengthen the basic knowledge on *L. monocytogenes* which may aid in understanding the mechanism of bacterial pathogenicity and virulence.

## Figures and Tables

**Table 1.** The physicochemical properties of the NP_464414.1 protein estimated by ProtParam

Descriptions	Value
No. of amino acids	115
Molecular weight (Da)	12,759.85
Theoretical PI	6.37
No. of positively charged residues	16
No. of negatively charged residues	16
No. of atoms	1839
Instability Index	36.52
Aliphatic Index	110.96
Grand average of hydropathicity	–0.057

**Table 2.** The subcellular localization prediction of the query protein NP_464414.1

Server	Final prediction
CELLO v.2.5	Cytoplasmic localization
PSORTb	Cytoplasmic localization
SOUSIGramN	Cytoplasmic localization
PSLpred	Cytoplasmic protein

**Table 3.** Result of CDD of NP_464414.1

Name	Acession	Description	Interval	E-value
PemK toxin	pfam02452	PemK-like, MazF-like toxin of type II toxin-antitoxin system	5–110	2.87e-43

**Table 4.** BLASTp result showing similarity between proteins

Accession No.	Organism	Protein name	Score	Protein identity (%)	E-value
NP_464414.1	*Listeria monocytogenes*	Hypothetical Protein			
WP_010989608.1	*Listeria monocytogenes*	Type II toxin-antitoxin system PemK/MazF family toxin	232	100	7.00E-77
WP_070005577.1	*Listeria monocytogenes*	Type II toxin-antitoxin system PemK/MazF family toxin	230	98.26	4.00E-76
WP_185340554.1	*Listeria seeligeri*	Type II toxin-antitoxin system PemK/MazF family toxin	229	97.39	2.00E-75
WP_046326403.1	*Listeria seeligeri*	Type II toxin-antitoxin system PemK/MazF family toxin	228	97.39	4.00E-75
EAF6615127.1	*Listeria monocytogenes*	Type II toxin-antitoxin system PemK/MazF family toxin	227	96.52	8.00E-75
WP_025279828.1	*Listeria ivanovii*	Type II toxin-antitoxin system PemK/MazF family toxin	226	96.52	2.00E-74
WP_003761304.1	*Listeria immobilis*	Type II toxin-antitoxin system PemK/MazF family toxin	226	97.37	2.00E-74
EFS00786.1	*Listeria seeligeri FSL N1-067*	Type II toxin-antitoxin system PemK/MazF family toxin	226	97.37	2.00E-74
EFR97581.1	*Listeria ivanovii FSL F6-596*	Type II toxin-antitoxin system PemK/MazF family toxin	224	96.49	1.00E-73

**Table 5.** Ramachandran plot statistics of the target protein

Statistics	No. of AA residues (%)
Residues in the most favored regions [A,B,L]	94 (92.2)
Residues in additional allowed regions [a,b,I,p]	7 (6.9)
Residues in generously allowed regions [⁓a, ⁓b,⁓l,⁓p]	1 (1)
Residues in disallowed regions	0 (0)
	Total (100)
No. of non-glycine and non-proline residues	102
No. of end residues (excl. Gly and Pro)	2
No. of glycine residues (shown in triangles)	7
No. of proline residues	4
Total No. of residues	115
